# Supplementary material for: Continuous sterane and phytane δ13C record reveals a substantial pCO2 decline since the mid-Miocene
Source: Nat Commun. 2024 Jun 18;15:5192. doi: 10.1038/s41467-024-47676-9 (PMC11189397; doi:10.1038/s41467-024-47676-9)
Supplement: Supplementary file 1 — Supplementary Information [file 41467_2024_47676_MOESM1_ESM.pdf]

Continuous sterane and phytane $\delta^{13}C$ record reveals a substantial pCO_2 decline since the mid-Miocene

Caitlyn R. Witkowski[1,2*], Anna S. von der Heydt[3], Paul J. Valdes[4], Marcel T.J. van der Meer[1], Stefan Schouten[1,5], Jaap S. Sinninghe Damsté[1,5]

[1]Department of Marine Microbiology and Biogeochemistry, NIOZ Royal Netherlands Institute for Sea Research, Den Burg (Texel), 1790AB, The Netherlands

[2]Schools of Earth Science and Chemistry, and the Cabot Institute, University of Bristol, Bristol, BS8 1RJ, UK

[3]Institute for Marine and Atmospheric Research Utrecht, Department of Physics, Utrecht University, Utrecht, 3584CC, The Netherlands

[4]School of Geographical Sciences and Cabot Institute, University of Bristol, Bristol, BS8 1SS, UK

[5]Department of Geosciences, Utrecht University, Utrecht, 3508 TA, The Netherlands

*Corresponding author: caitlyn.witkowski@bristol.ac.uk

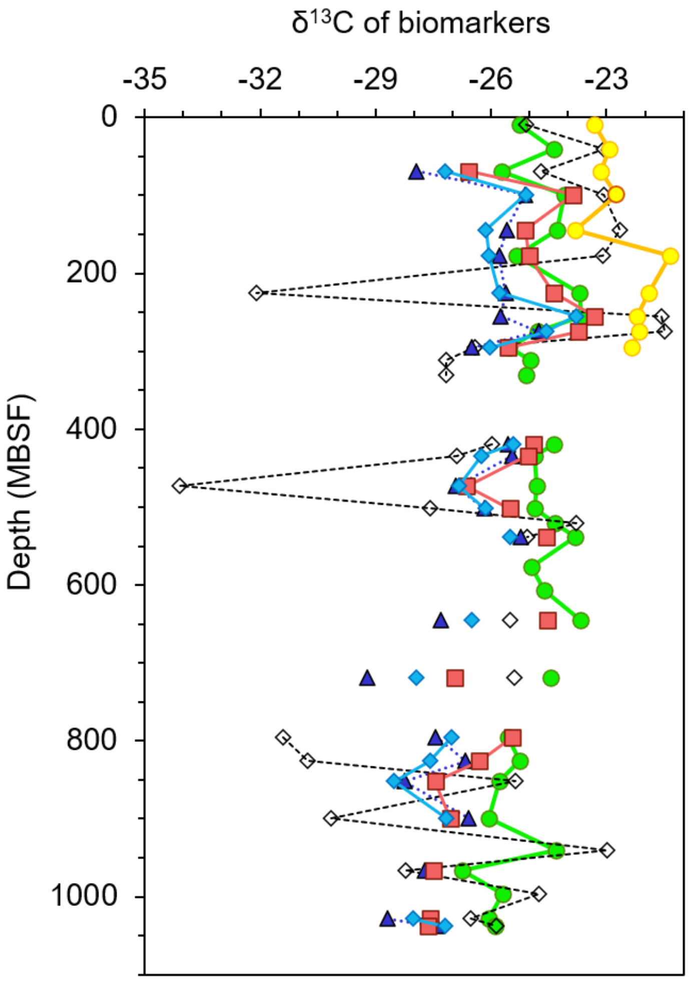

Fig. S1. $\delta^{13}C$ records of 5α-cholestane (red square), 24-ethyl-5α-cholestane (cyan diamond), 24-methyl-5α-cholestane (blue triangle), phytane (green circle), alkenones (yellow circle), and C_{25} highly branched isoprenoids (white diamond) used in this study at the mid-latitude DSDP Site 467. C_{25} HBIs are considered a biomarker for diatoms, which are common upwelling species. The $\delta^{13}C$ of the C_{25} HBIs have very different values and trends than the other GPBs, suggesting this species (and by association, upwelling) likely play a minor role at this site.

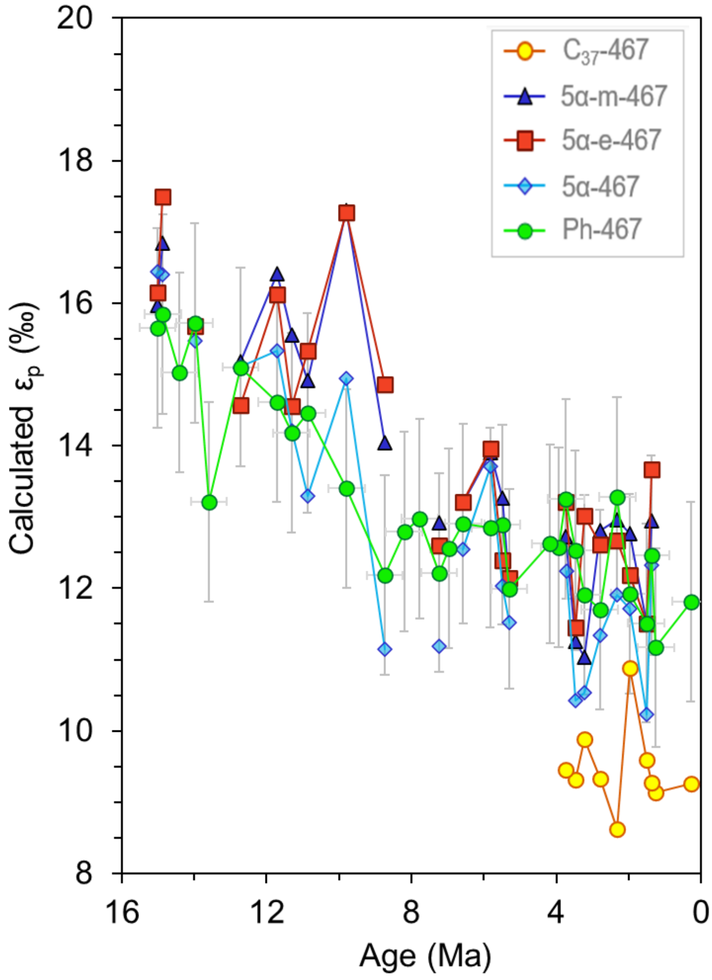

Fig. S2. Calculated ε_p over the past 15 Myr. ε_p based on the $\delta^{13}C$ records of 5α-cholestane (red square), 24-ethyl-5α-cholestane (cyan diamond), 24-methyl-5α-cholestane (dark blue triangle), phytane (green circle), and alkenones (yellow circle) at mid-latitude DSDP Site 467. Error bars on phytane are based on Monte Carlo simulations, incorporating one standard deviation uncertainty on compounding all input parameters.[9]

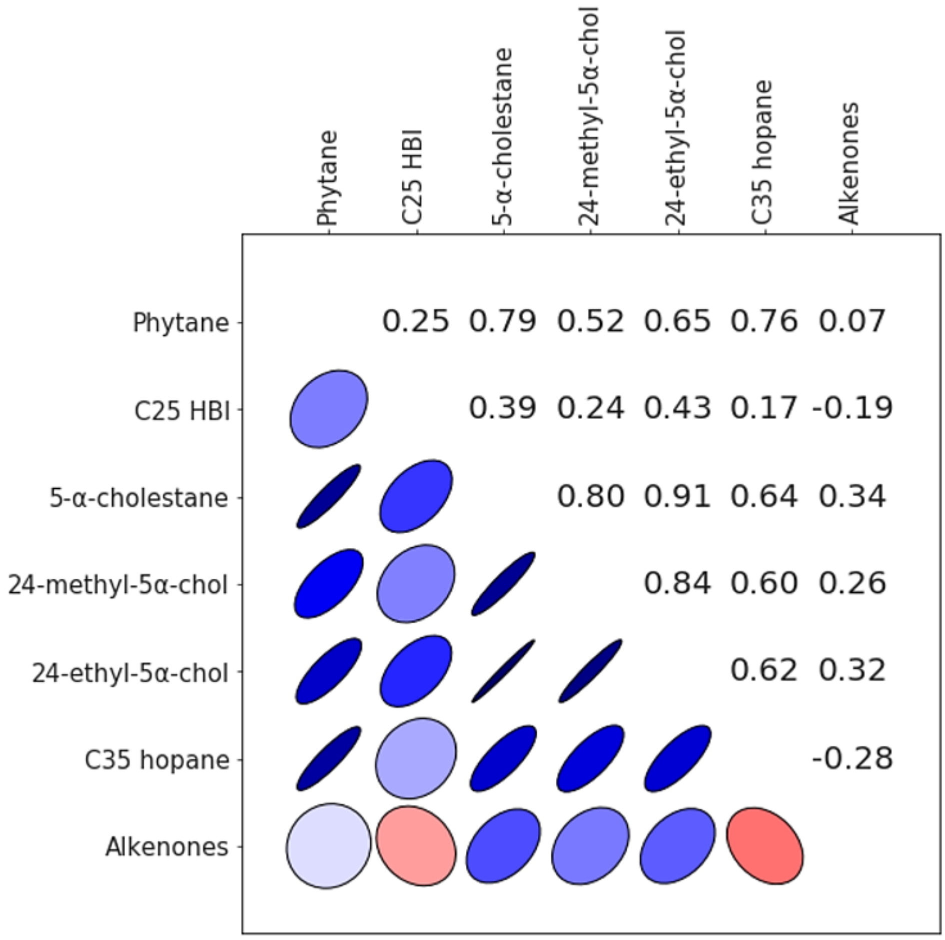

Fig. S3. Cross-correlation plot of the $\delta^{13}C$ for the examined biomarkers: 5α-cholestane, 24-methyl-5α-cholestane, 24-ethyl-5α-cholestane, phytane, and alkenones used in this study at the mid-latitude DSDP Site 467. Additionally, we have also included the diatom-derived C_{25} highly branched isoprenoid (2,6,10,14-tetramethyl-7 (3'-methylpentyl)pentadecane)) which suggests a lack of upwelling. Blue shows positive correlation, where increasing darkness and decreasing width visualize stronger correlations. Red indicates negative correlation.

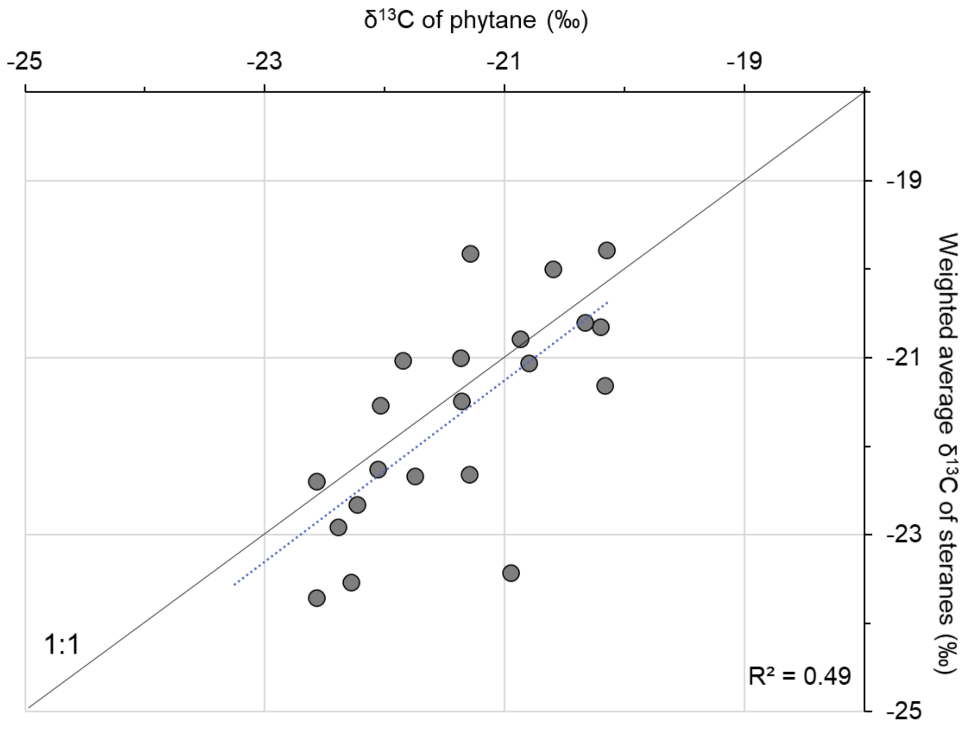

Fig. S4. Cross-plot of the δ^{13}C of phytane versus steranes. Weighted sterane average is based on the fractional abundance of 5α-cholestane, 24-methyl-5α-cholestane, and 24-ethyl-5α-cholestane.

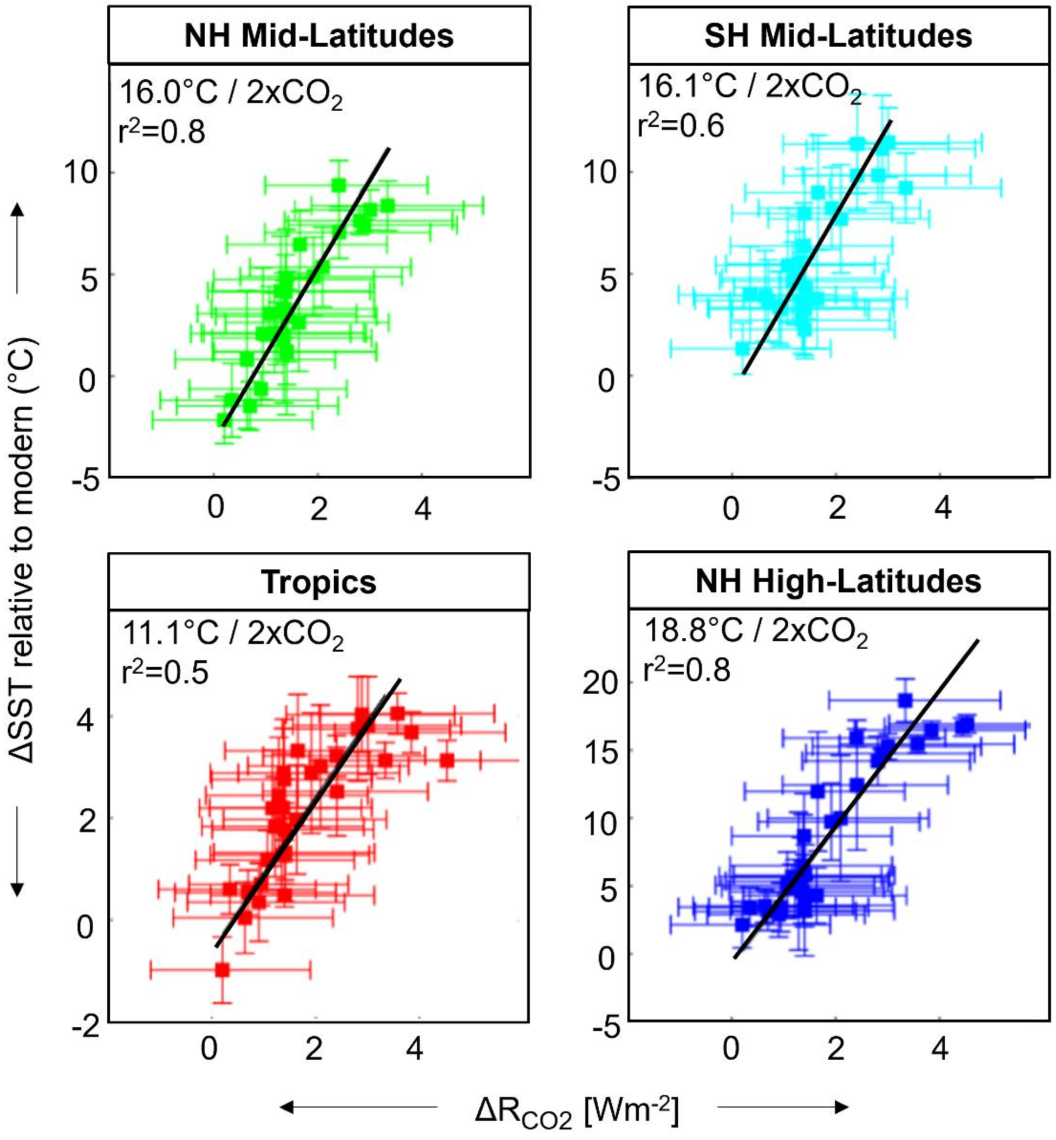

Fig. S5. Relationship between temperature and pCO$_2$ from 15.0–0.3 Ma. Y-axis: $U^{K'}_{37}$-based sea surface temperature (SST) changes relative to the modern mean annual SST at each site[10], labeled: northern hemisphere (NH) mid-latitudes (green), southern hemisphere (SH) mid-latitudes (cyan), tropics (red), and NH high latitudes (blue). *Note: Tropics $U^{K'}_{37}$ ratio approaching saturation beyond 8 Ma cannot be used for a good computation of climate sensitivity, thus data >8 Ma (open black squares) are plotted but removed from fit. X-axis: Radiative forcing due to CO_2 (ΔR_{CO2}), where CO_2 estimations are derived from the GPB phytane. Top left corners show Earth system sensitivity (ESS) change in temperature per doubling of CO_2 based on the slope of linear fit of data with r^2 values.
