# Supplementary material for: Continuous sterane and phytane δ13C record reveals a substantial pCO2 decline since the mid-Miocene
Source: Nat Commun. 2024 Jun 18;15:5192. doi: 10.1038/s41467-024-47676-9 (PMC11189397; doi:10.1038/s41467-024-47676-9)
Supplement: Supplementary file 3 — Description of Additional Supplementary Files [file 41467_2024_47676_MOESM3_ESM.pdf]

Description of Additional Supplementary Files:

Supplementary Data 1: All data and uncertainties used to estimate *p*CO_2 from the $\delta^{13}C$ of phytane.

Supplementary Data 2: All data and uncertainties used to estimate *p*CO_2 from the weighted average of the $\delta^{13}C$ of steranes based on their fractional abundances (i.e., 5α-cholestane, 24-ethyl-5α-cholestane, and 24- methyl-5α-cholestane).

Supplementary Data 3: All data and uncertainties used to estimate *p*CO_2 from the $\delta^{13}C$ of 5α-cholestane.

Supplementary Data 4: All data and uncertainties used to estimate *p*CO_2 from the $\delta^{13}C$ of 24-ethyl-5α-cholestane.

Supplementary Data 5: All data and uncertainties used to estimate *p*CO_2 from the $\delta^{13}C$ of 24-methyl-5αcholestane.

Supplementary 6: All data and uncertainties used to estimate *p*CO_2 from the $\delta^{13}C$ of alkenones.

Supplementary Data 7: All data and uncertainties used to estimate *p*CO_2 from the $\delta^{13}C$ of phytol from the literature.

Supplementary Data 8: All raw data from DSDP Leg 63 Site 467, including total organic carbon percentage, $\delta^{13}C$ values of algal biomarkers (phytane, steranes, C_{25} HBIs, C_{35} hopane, alkenones), and GDGT abundances used to calculate sea surface temperatures using TEX_{86}.

Supplementary Data 9: Revised age model from DSDP Leg 63 Site 467.
